# A systematic review and meta-analysis: the therapeutic and preventive effect of Lactobacillus reuteri DSM 17,938 addition in children with diarrhea

**DOI:** 10.1186/s12876-023-02778-4

**Published:** 2023-05-05

**Authors:** Xiaoqi Sun, Juan Kong, Shuotong Zhu, Chengjiang Liu

**Affiliations:** 1 Department of Clinical Nutrition, Shengjing Hospital of China Medical University, Shenyang, China; 2 Department of Gerneral Surgery, Fourth Affiliated Hospital of China Medical University, Shenyang, China; 3 Department of Gastroenterology, Anhui Medical University, He Fei, China

**Keywords:** Lactobacillus reuteri, Diarrhea, Probiotics, Clinical response

## Abstract

**Objective:**

To summarize the effect of adding Lactobacillus reuteri in the treatment plan for diarrheal disease in children, and analyze the potential of probiotics in preventing the occurrence of diarrheal disease.

**Methods:**

Search for randomized controlled trials of Lactobacillus reuteri for the treatment and prevention of diarrhea in the Pubmed, Web of science, Medline, and Cochrane databases. Data such as the number of diarrhea patients, time, length of stay, clinical symptoms and effect of diarrhea prevention were extracted for meta-analysis. Relative risk and confidence interval (RR and 95% CI) were used as outcome indicators.

**Results:**

963 participants in the 9 RCTs came from multiple countries/regions. Compared with placebo/no intervention, the number of diarrhea patients in the Lactobacillus reuteri group was significantly reduced on the day 1 (RR = 0.87, 95%CI: 0.78–0.97) and day 2 (RR = 0.61, 95%CI: 0.44–0.83). Cumulative statistics analysis showed that the effect was stable and significant starting on the 4th day after treatment. A few studies have shown that Lactobacillus reuteri can reduce the time of diarrhea, the number of days with watery stools, and days of hospital stay. However, it has no effect on the occurrence of nosocomial diarrhea (RR = 1.11, 95%CI: 0.68–1.83), rotavirus diarrhea (RR = 1.46, 95%CI: 0.78–2.72), antibiotic-related diarrhea (RR = 1.76, 95%CI: 0.77–4.05), and diarrhea (RR = 1.35, 95%CI: 0.95–1.92).

**Conclusion:**

Addition of Lactobacillus reuteri in the treatment plan has a significant effect on reducing the number of diarrhea and reducing the symptoms of diarrhea, but has no obvious effect on the prevention of diarrhea. Combining probiotics and improving the ability of probiotics to respond is the focus of attention.

## Background

Diarrheal diseases are the second most common cause of death and leading cause of death among children in the world [1, 2]. Diarrhea was usually defined as 3 or more loose or watery stools, or 1 or more bloody stools in 24 h [3]. Diarrhea symptoms typically lasts less than 7 days, not longer than 14 days [4]. Evidence-based guidelines for the management of acute gastroenteritis indicate that fluid replacement is the key treatment method. It also shows that probiotics can reduce the duration and intensity of symptoms, and can be used as an adjuvant for oral rehydration solutions (ORS) [5]. Although rotavirus vaccination has been introduced in many countries recently, the burden of diarrhea has not been eliminated because of this form of primary prevention. Consequently, the role of probiotics in the treatment of diarrheal diseases was still widely concerned.

The efficacy and safety of probiotics need to be determined due to the specificity of the strain. Previous prospective randomized trials have showed Lactobacillus reuteri ATCC 55,730 (L. reuteri) was proven to colonize the gastrointestinal tract effectively and shorten the duration of watery diarrhea associated with rotavirus infection time significantly [6]. Lactobacillus reuteri DSM 17,938 is a gram-positive bacterium that naturally exists in the intestinal tract of mammals. It loses the abnormal and transferable resistance of the tetracycline and lincomycin it carries by removing two plasmids [7]. The key to treat children's diarrhea effectively depend on the production of the anti-pathogenic compound reuterin and immunomodulatory ability. Moreover, which need to aggregate and co-aggregate helps to colonize the gastrointestinal tract and eliminate pathogens from it [8, 9].

Recently, new evidence has emerged regarding the effectiveness of Lactobacillus reuteri DSM 17,938. Compared with placebo, Lactobacillus reuteri DSM 17,938 can shorten the hospital stay of children under 5 years of age with AGE other than the duration of diarrhea as an adjunct to rehydration therapy [10]. However, this is contrary to the conclusion of a previous systematic review, and it is not clear whether the addition of Lactobacillus reuteri can prevent diarrhea [11]. Our aim was to update data on the ability of Lactobacillus reuteri DSM 17,938 to treat and prevent various types of diarrheal diseases in children.

## Material and methods

### Search strategy

Pubmed, Web of Science, Medline, Cochrane library databases were searched for eligiity publications. Two researchers designed and implemented this search strategy. The publication time was limited until June 2021. P(Children suffering from diarrhoea or preventive healthy children), I(Addition of Lactobacillus reuteri in general treatment plan), C(Placebo), O(Number of diarrhea cases, severity score of diarrhea, duration of diarrhea, days of water sample defecation, average hospitalization days, etc.). Following keywords were used: (Lactobacillus reuteri OR L. reuteri OR Lactobacillus OR probiotic*) AND (diarrhea OR diarrhoea OR diarrh*). Title and abstract of the article were checked to filter, and the full text was obtained. We also manually screen the references of retrieved articles to identify other relevant studies.

### Inclusion and exclusion criteria

Research is considered available if the publication meets all of the following criteria:

Confirm the diagnosis as diarrhea and Research is considered available if the publication meets all of the following criteria:

1. Confirm the diagnosis as diarrhea and identify similar diagnoses;
2. Randomized clinical trial;
3. A detailed and accurate description of the experiment participants (children and healthy people);
4. A complete and appropriate result description;
5. Follow-up time was long enough for the expected outcome.

The criteria for exclusion are as follows:

1. Unreliable or inaccurate disease diagnosis;
2. Comments, abstracts, editorials;
3. Animal tissue research;
4. Research that does not provide sufficient data.

### Risk of bias for included studies

The two evaluators independently evaluated the test quality and bias risk according to the tools of Cochrane collaboration network [12]. Possible differences shall be resolved by the third reviewer or consensus based discussion. Included items were followed: randomization methods, allocation hiding methods, blinding of participants and implementers, blinding of result evaluation, and incomplete result data. In addition, selective reporting and other types of bias are also considered. If it can’t be assessed due to missing information, we rate the corresponding item as an unclear risk of bias.

### Data extraction

Two evaluators independently extracted data according to the pre-strategy. The following information was extracted: title; author's name; publication year; study design; objective; number of participants; intervention plan and time; primary results (diarrhea cases), secondary indicators (diarrhea severity score, diarrhea duration, water samples Days of defecation, average days of hospitalization, days lost in day care, days of parental care). Disagreements arising during the extraction process shall be resolved by the third reviewer or the original author through email.

### Statistical analysis

Data was analyzed using Stata 16.0 software (StatCorp, USA). The measurement data is expressed by the mean ± sd. RR is used as the main statistic in this study. Heterogeneity test was used I^2^ statistic. If I^2^ values > 50% indicate that heterogeneity was observed among studies and the random effects model was applied. Fixed effects model was applied when there is no heterogeneity among studies. Sensitivity analysis was achieved by excluding study one by one and examining the impact of each study on comprehensive RR. Funnel plots were used to detect whether there is a small research effect. The publication bias was comprehensively evaluated by Begg's test and Egger's test.

## Results

### Basic characteristic of included studies

**Fig. 1:**
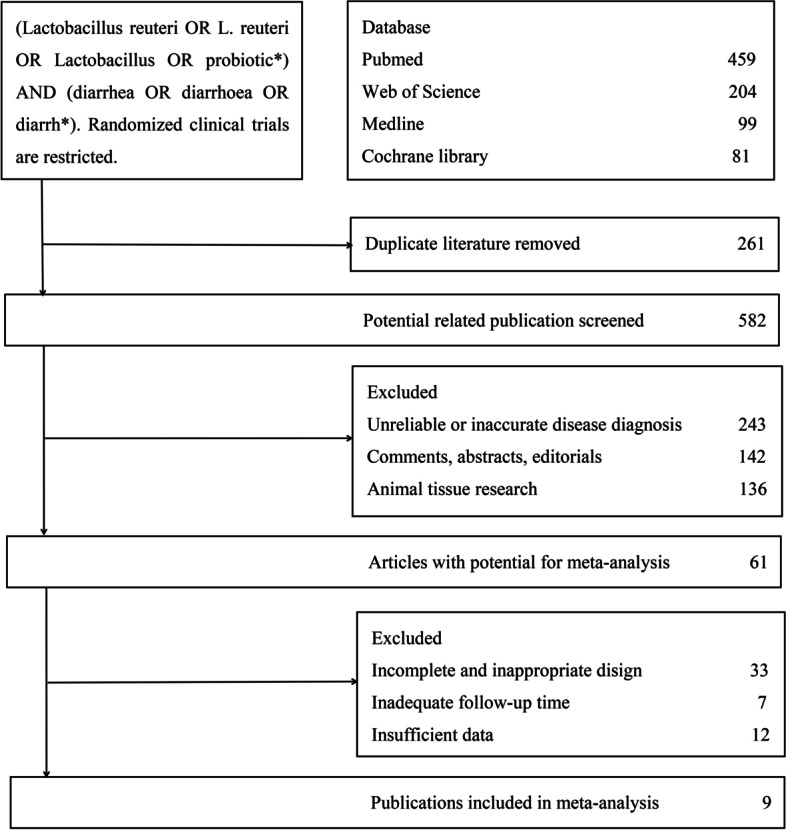
Screening flowchart for systematic reviews and meta-analysis

**Fig. 2:**
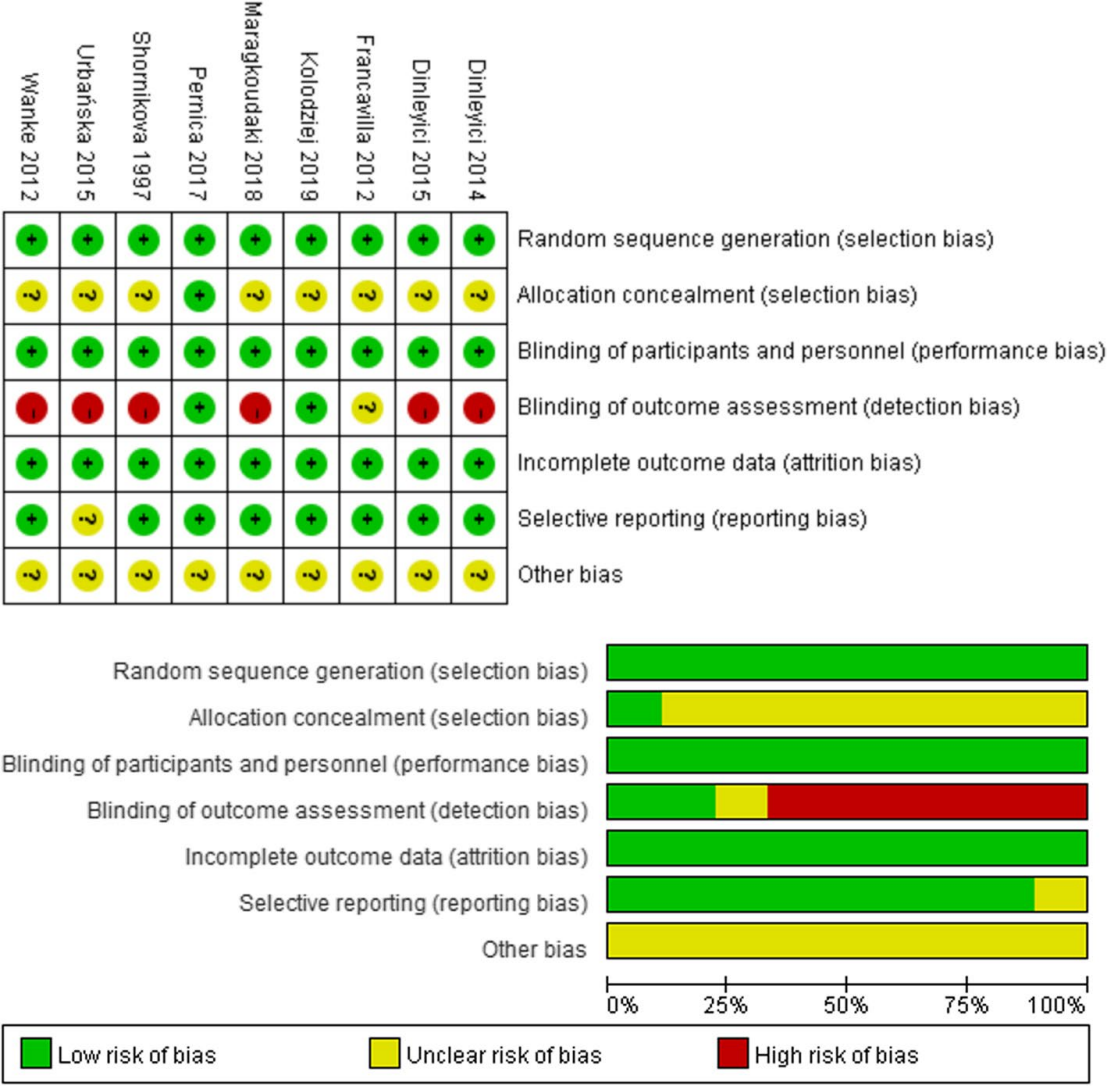
Diagram of risk bias of included studies

Based on the pre-screening strategy, two researchers finally selected 9 randomized clinical trials (6 treatment trials for diarrhea in children, 3 prevention trials for diarrhea in healthy children) [13–21]. The screening process was summarized in Fig. 1. 963 participants from many countries/regions in the world were selected firstly. Participants were under 60 months, and the experimental group and the control group had good comparability in demographic characteristics. Dosage of Lactobacillus reuteri in the treatment plan of the experimental group was more than 10 (8) CFU/daily. Choose an appropriate placebo-controlled regimen and continue the intervention for no less than 5 days. Neither the experimenter nor the participants knew the allocation method. Table 1 listed the characteristics of these studies. In Fig. 2, the risk bias diagram showed that some of the studies [13–15, 18–20] were at high risk. For most studies, the allocation bias and other biases were not clear. Other projects with low risk indicated that these studies are suitable for inclusion in meta-analyses.

**Table 1:** Basic characteristic of included studies

Study	Country	Research design	Objective	Sample(Exp VS. Con)	Age(Year)	Gender(Male/Female)	Intervention	Outcome
Maragkoudaki 2018 [13]	Greece	RCT,double-blind	Treatment	28	1.7 ± 0.7 years	21/7	10(8)CFU + ORS,5 days	No diarrhea(13/28, day 1), (18/28, day 2), (20/28, day 3), (19/28, day 4), (26/28, day 5); Watery stool(1.6, 1.2–2.0)days; Soft stool(4.0, 3.2–4.8)days
				23	1.8 ± 0.7 years	16/7	Placebo(ORS)	No diarrhea (12/23,day 1), (13/23,day 2), (13/23,day 3), (17/28,day 4), (16/23,day 5); Watery stool(2.1,1.4–2.7)days; Soft stool(4.5, 3.5–5.5)days
Shornikova 1997 [6, 14]	Finland	RCT,double-blind	Treatment	19	16.8 ± 8.1momths	NA	10(10)-10(11)CFU daily,5 days	Diarrhea(16, day 1), (5, day 2), (2, day 3), (3, day 4), (2, day 5), (2, day 6); Watery stool(1.7 ± 1.6)days
				21	16.3 ± 9.5 months	NA	Placebo (nonfat dry milk powder)	Diarrhea(21, day 1), (17, day 2), (11, day 3), (6, day 4), (3, day 5), (3, day 6); Watery stool(2.9 ± 2.3)days
Dinleyici 2015 [15]	Turkey	RCT,single-blind	Treatment	29	27.9 ± 18.2 months	20/9	10(8)CFU + ORS,5 days	Diarrhea: (OR = 0.86,0.74–1.01, day 1), (OR = 0.51,0.34–0.79, day 2), (OR = 1.34,0.40–4.50, day 3); duration of diarrhea (60.4 ± 24.5 h)
				31	22.6 ± 14.4 months	22/9	Placebo(ORS)	Duration of diarrhea (74.3 ± 15.3 h)
Francavilla 2012 [16]	Italy	RCT,double-blind	Treatment	35	26.1 ± 4.1 months	23/14	4 × 10(8)CFU daily;7 days	Diarrhea(31, day 1), (19, day 2), (16, day 3), (14, day 4), (9, day 5), (2, day 6), (0, day 7); Watery stool (2.1 ± 1.7 days); Recurrence rate: 15%
				34	25.4 ± 2.1 months	22/15	Placebo (mixture of sunflower oil and medium-chain triglyceride oil)	Diarrhea(34, day 1), (28, day 2), (25, day 3), (17, day 4), (11, day 5), (3, day 6), (1, day 7); Watery stool (3.3 ± 2.1 days); Recurrence rate: 42%
Pernica 2017 [17]	Botswana	RCT,triple-blind	Treatment	18	0.98 ± 0.71 years	13/5	1 × 10(8)CFU daily;60 days	Recurrence rate(OR = 0.07, 0.01–0.61)
				17	1.11 ± 0.76 years	8/9	Placebo	
	Botswana	RCT,triple-blind	Treatment	16	1.07 ± 0.49 years	7/9	1 × 10(8)CFU daily;60 days	
				20	1.21 ± 0.89 years	11/9	Placebo	
Dinleyici 2014 [18, 22]	Turkey	RCT,single-blind	Treatment	64	3-60 months	NA	10(8)CFU daily,5 days	No diarrhea(50%, day 2), (69%, day 3)
				63	3-60 months	NA	Placebo	No diarrhea(5%, day 2), (11%, day 3)
Wanke 2012 [19]	Poland	RCT,double-blind	Prevention	54	1-48 months	NA	10(8)CFU once daily	Nosocomial diarrhea (RR = 1.06, 0.7–1.5); rotavirus diarrhea (RR = 1.04, 0.6–1.6); diarrhea (RR = 1.26, 0.75–2.14)
				52	1-48 months	NA	Placebo	
Urbańska 2016 [20]	Poland	RCT,double-blind	Prevention	91	1-48 months	NA	10(9)CFU daily	Nosocomial diarrhea(7 vs 6); rotavirus diarrhea (RR = 3.07, 0.13–74.28); diarrhea (RR = 1.12, 0.50–2.52)
				93	1-48 months	NA	Placebo (maltodextrin)	
Kolodziej 2019 [21]	Poland	RCT,triple-blind	Prevention	125	25.7 ± 35.2 months	72/53	2 × 10(8)CFU twice daily;2 weeks	Rotavirus diarrhea (RR = 2.02, 0.71–5.73); diarrhea (RR = 1.58, 0.89–2.80); antibiotic-associated diarrhea (RR = 1.76, 0.77–4.05)
				125	25.8 ± 33.8 months	71/54	Placebo(mixture medium-chain triglycerides, sunflower oil and silicon dioxide)	

*RCT* Randomized controlled trial, *NA* Not available, *ORS* Oral rehydration salts

### Improvement of diarrhea with Lactobacillus reuteri alone or in combination

**Fig. 3:**
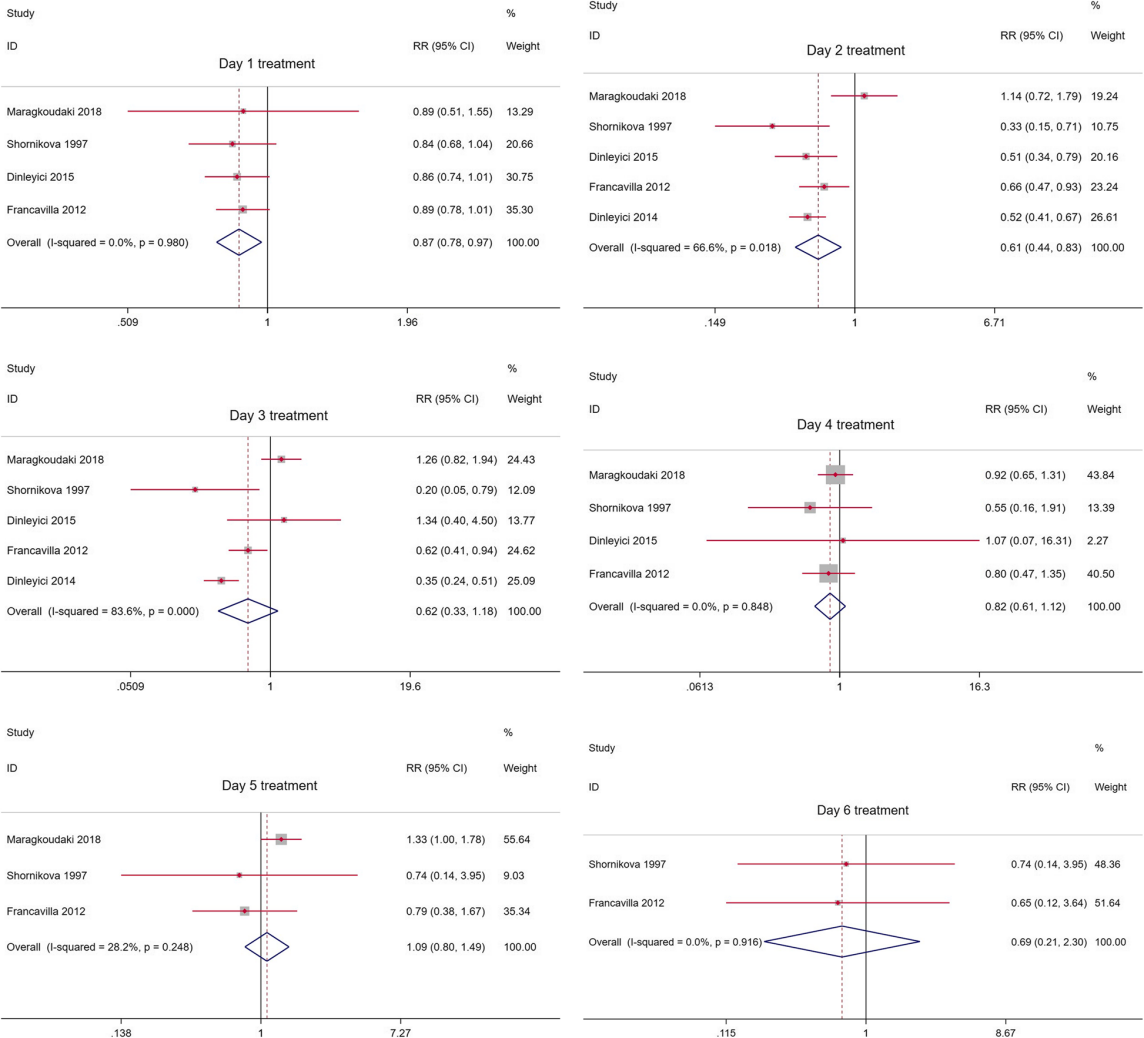
Number of diarrhea cases with Lactobacillus reuteri treatment compared to placebo/no intervention (day 1–6)

6 RCTs are based on the improvement in the number of diarrhea cases of Lactobacillus reuteri compared with placebo treatment as the main evaluation index. If the I^2^ statistic is less than 50%, the fixed effects model is used to fit the total RR, otherwise the random effects model. The number of diarrhea in the Lactobacillus reuteri group was significantly reduced on day 1 (RR = 0.87, 95%CI: 0.78–0.97, I^2^ = 0%, *p* = 0.980, Fig. 3) and day 2 (RR = 0.61, 95%CI: 0.44–0.83, I^2^ = 66.6%, *p* = 0.018, Fig. 3). On day 3 (RR = 0.62, 95%CI: 0.33–1.18, I2 = 83.6%, *p* = 0, Fig. 3), day 4 (RR = 0.82, 95%CI: 0.61–1.12, I^2^ = 0%, *p* = 0.848, Fig. 3), day 5 (RR = 1.09, 95%CI: 0.80–1.49, I^2^ = 28.2%, *p* = 0.248, Fig. 3), day 6 (RR = 0.69, 95%CI: 0.21–2.30, I^2^ = 0%, *p* = 0.916, Fig. 3) showed no significant difference. Excluding individual studies successively, no significant changes in statistics were found, which shows the stability of the results. Neither Begg's test nor Egger's test found publication bias (*p* > 0.05).

**Fig. 4:**
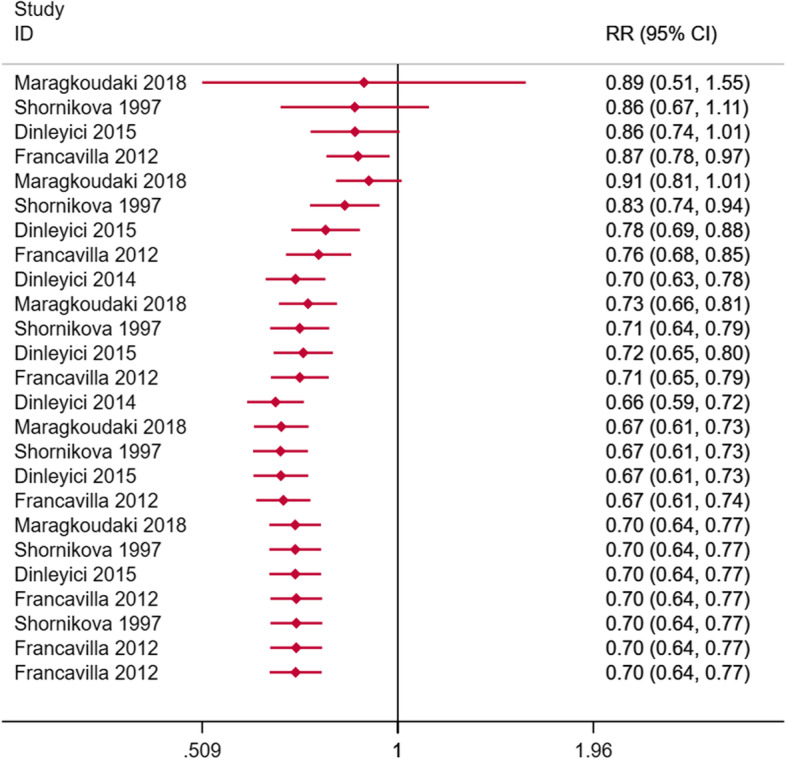
Cumulative RRs based on treatment days using a fixed effect model

**Fig. 5:**
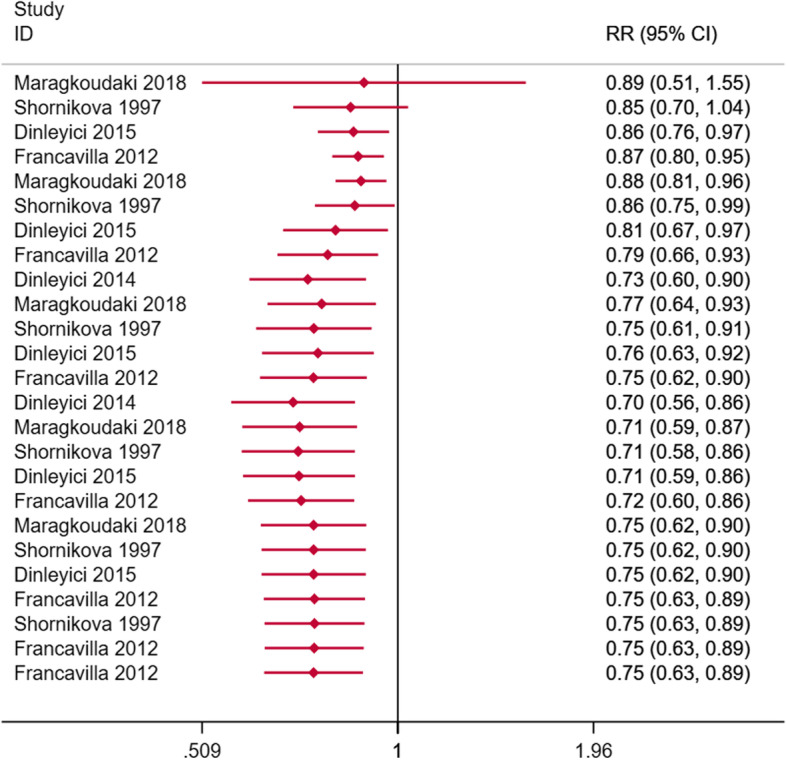
Cumulative RRs based on treatment days using a random effect model

We respectively used a fixed-effect model and a random-effect model to fit the cumulative improvement of diarrhea during Lactobacillus reuteri treatment (Figs. 4, 5). It can be seen that Lactobacillus reuteri did not improve significantly at the initial stage of treatment (day 1), and the treatment effect became stable after entering the medication process (days 3–7), showing a clear J-shaped trend, and long-term trends are improving.

### The preventive effect of Lactobacillus reuteri on diarrhea

**Fig. 6:**
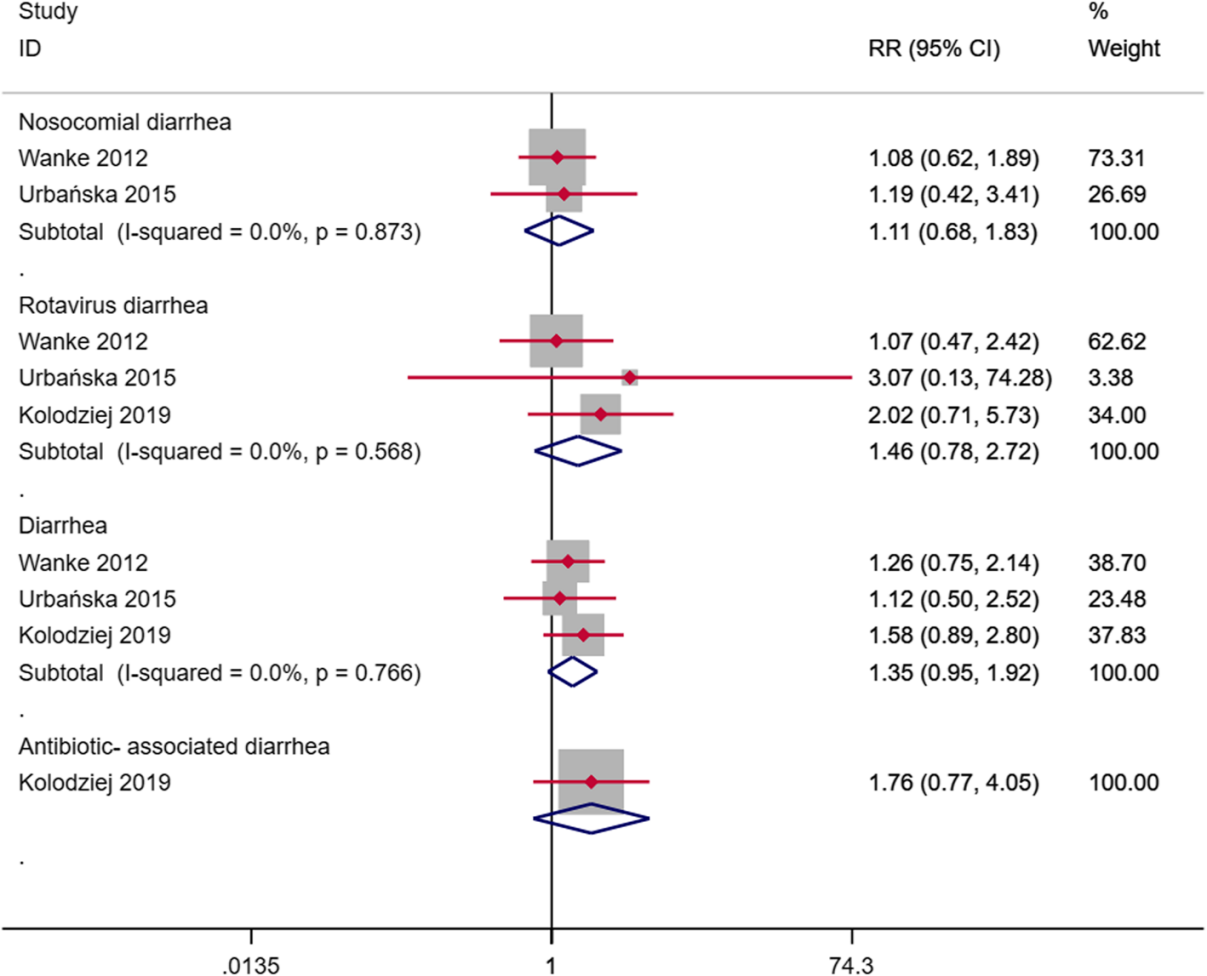
RRs that added Lactobacillus reuteri to prevent nosocomial diarrhea, rotavirus diarrhea, diarrhea, and antibiotic-related diarrhea compared to placebo

3 studies reported the preventive effect of Lactobacillus reuteri in hospital diarrhea. Lactobacillus reuteri had no significant preventive effect to nosocomial diarrhea (RR = 1.11, 95%CI: 0.68–1.83, I^2^ = 0%, *p* = 0.873, Fig. 6), rotavirus diarrhea (RR = 1.46, 95%CI: 0.78–2.72, I^2^ = 0%, *p* = 0.568, Fig. 6), diarrhea (RR = 1.35, 95%CI: 0.95–1.92, I^2^ = 0%, *p* = 0.766, Fig. 6), antibiotic-related diarrhea (RR = 1.76, 95%CI: 0.77–4.05, Fig. 6). Excluding individual studies successively, no significant changes in statistics were found, which shows the stability of the results. Begg's test and Egger's test did not find publication bias (*p* > 0.05).

### Secondary effects of Lactobacillus reuteri in diarrhea

Diarrheal diseases often bring losses to individuals and family members. In Maragkoudaki 's study, compared with placebo, the treatment with Lactobacillus reuteri reduced the number of days of watery stool by 2.1 days (95%CI: 1.4–2.7 days) vs. 1.6 days (95%CI: 1.2- 2.0 days), and 4.5 days (95%CI: 3.5–5.5 days) vs. 4.0 days (95%CI: 3.2–4.8 days) with soft stools. The same reduced the number of day care days lost due to diarrhea by 3.0 days (95%CI: 1.9–4.1 days) vs. 1.8 days (95%CI: 0.6–3.0 days), and the number of days of parental care 1.4 days (95%CI: 0.5) -2.3 days) vs. 1.1 days (95%CI: 0.4–1.8 days). Dinleyici's 2014 study found that treatment with Lactobacillus reuteri reduced the average hospital stay from 5.46 ± 1.77 days to 4.31 ± 1.3 days. Dinleyici's 2015 study found that Lactobacillus reuteri significantly reduced the duration of diarrhea from 74.3 ± 15.3 h to 60.4 ± 24.5 h. In the Francavilla 2012 study, Lactobacillus reuteri significantly reduced the duration of watery diarrhea (2.1 ± 1.7 days vs. 3.3 ± 2.1 days). The recurrence rate of diarrhea in children treated with Lactobacillus reuteri was significantly lower (15% vs. 42%). Pernica's 2017 study found that the combination of rapid testing and treatment with Lactobacillus reuteri treatment resulted in a 60-day normalized height (HAZ) associated with a statistically significant increase of 0.61, with a lower chance of recurring diarrhea during the follow-up period (OR, 0.07). The use of Lactobacillus reuteri alone (that is, no rapid testing and treatment) was associated with the probability of recurrent diarrhea (OR, 0.10) during the 60-day follow-up period and a non-significant increase in the 60-day HAZ 0.51.

## Discussion

### Findings

Our evaluation confirmed that in the treatment of diarrhea, compared with placebo or control, on the first day of adding Lactobacillus reuteri DSM 17,938 (RR = 0.87, 95%CI: 0.78–0.97, I^2^ = 0%, *p* = 0.980, Fig. 3) and the next day (RR = 0.61, 95%CI: 0.44–0.83, I^2^ = 66.6%, *p* = 0.018, Fig. 3) can significantly reduce the number of patients. However, the effect is not significant in the next few days. The gradual decrease in the efficacy of the drug exhibits a J-shaped trend. Therefore, we try to perform a cumulative fitting of statistics based on the number of days of treatment. It was found that Lactobacillus reuteri had a stable and significant improvement effect after about 4 days of treatment. We also checked less researched diarrhea-related indicators such as diarrhea severity score, days of watery stools, days of soft stools, average days of hospitalization, days lost in day care, and days of parental care. Addition of Lactobacillus reuteri will have different degrees of improvement compared with placebo and no intervention. Experiments conducted in the hospital to prevent diarrhea diseases did not find that Lactobacillus reuteri could reduce the incidence of hospital diarrhea, rotavirus diarrhea, diarrhea, and antibiotic-related diarrhea, which may not be in line with our expectations. It is still reasonable considering the number of participants and the conduct in the hospital. It is worth noting that no adverse events have been observed.

### Agreement and disagreement with other studies or reviews

Previously, two studies found significant healing effects only in the first 2 days of Lactobacillus reuteri treatment [23, 24]. Our research not only further confirmed, but also found that starting from the 4th day of treatment, the healing effect tends to be significant and stable compared with placebo or no intervention. Considering the quantity and quality of the included literature, our results are more trustworthy. A systematic review of randomized placebo-controlled trials of Lactobacillus reuteri DSM 17,938 found that this probiotic effectively reduced the duration of diarrhea and hospitalization [24]. Although this effect was observed when only studies with sufficient blinding and allocation concealment were analyzed Smaller. This conclusion was confirmed in our research. An in vitro experiment found that L. reuteri LMG P-27481 strain is a very effective probiotic candidate for the treatment of CD infection [25]. This is actually different from our research. Due to the specificity of the strain, this possibility does exist.

Research on other probiotics has also received attention. A recent analysis of data from 5 high-quality RCTs (with no or only one area of unclear risk of bias) showed that Lactobacillus rhamnosus had no effect on the duration of diarrhea (MD = -0.68, 95% CI:-1.81–0.44) days [26]. Three systematic reviews [22, 27, 28] consistently reported that the use of saccharomyces boulardii significantly shortened the duration of diarrhea compared with the placebo group or the no-intervention group, although their inclusion was not the same. As far as we know, there is no reported data that Lactobacillus reuteri DSM 17,938 causes any serious adverse events even when used in premature infants [29]. A variety of Lactobacillus strains together with Lactobacillus reuteri or Lactobacillus plantarum as therapeutic agents or nutritional supplements may be a new method to prevent and treat diarrhea in children. Evaluating the effectiveness and safety of various strains is of great significance for subsequent research. Highly adherent Lactobacillus reuteri shows higher benefits because it means higher cell membrane permeability and stronger mucin capacity [30]. Fortunately, we are consistent with the conclusions of the recent systematic review. In general, probiotics can be used safely in other healthy people [31].

### Strengths and limitations

We specifically evaluated the effectiveness of Lactobacillus reuteri DSM 17,938, but did not evaluate the effectiveness of its original strain Lactobacillus reuteri ATCC 55,730. Evaluation is based on a method developed in collaboration with Cochrane and reported in accordance with the PRISMA statement. Many efforts have been made to reduce the risk of bias (for example, no language or date restrictions are imposed). The risk of bias in the included trials was also evaluated based on the design of the study design. Finally, our research focuses on the application of a single probiotic that is available in many countries/regions, so the research results are applicable to practice. However, this evaluation has several important limitations. First, only a few studies are available. Second, some included trials have unclear or high risks of bias, which raises questions about the reliability of the results provided. Third, because there are fewer trials available, trials with mixed (unclear, high or low) levels of bias in certain areas are combined in the analysis. In some more limited studies, meta-analysis is not possible. Fourth, the significant heterogeneity between studies can only be partially explained by differences in research design methods. However, no subgroup meta-analysis was performed due to the small number of studies. However, previous studies of different probiotics have shown that the specific effects of probiotics on intestinal pathogens seem unlikely [32, 33]. Although this review evaluated the effectiveness of probiotics in preventing diarrhea in community trials. Due to the presence of other confounding factors, our findings may need to be confirmed in more experiments in different populations. Another limitation was the definition of diarrhea and the duration of diarrhea. Some results, the number of days in hospital due to diarrhea, the number of days lost in day care, the number of days of parental care, etc., have only been evaluated in a limited number of trials; therefore, these findings may only be accidental. Finally, due to lack of data, it is not possible to clearly assess the impact of Lactobacillus reuteri DSM 17,938 on stool volume and the severity of diarrhea score.

## Conclusion

We summarized the significant effect of adding Lactobacillus reuteri to the treatment regimen in reducing the number of diarrhea cases and reducing the symptoms of diarrhea compared with placebo or no intervention. Although Lactobacillus reuteri has not been found to be effective in preventing diarrhea, this may be caused by a variety of confounding factors due to the imprecise experimental design. More higher-quality RCT evidence is needed. Combining a variety of probiotics and improving the effect of probiotics in diseases will be the focus of research.

## Data Availability

The data in this article comes from public domain databases. The datasets used and/or analysed during the current study available from the corresponding author on reasonable request.

## References

[1] Bern C, Martines J, de Zoysa I, Glass RI (1992). The magnitude of the global problem of diarrhoeal disease: a ten-year update. Bull World Health Organ.

[2] Yip R, Sharp TW (1993). Acute malnutrition and high childhood mortality related to diarrhea. Lessons from the 1991 Kurdish refugee crisis. JAMA..

[3] DuPont HL (1997). Guidelines on acute infectious diarrhea in adults. The Practice Parameters Committee of the American College of Gastroenterology. Am J Gastroenterol..

[4] Guarino A, Albano F, Ashkenazi S, Gendrel D, Hoekstra JH, Shamir R, Szajewska H, European Society for Paediatric Gastroenterology, Hepatology, and Nutrition; European Society for Paediatric Infectious Diseases (2008). European Society for Paediatric Gastroenterology, Hepatology, and Nutrition/European Society for Paediatric Infectious Diseases evidence-based guidelines for the management of acute gastroenteritis in children in Europe. J Pediatr Gastroenterol Nutr.

[5] Guarino A, Albano F, Ashkenazi S, Gendrel D, Hoekstra JH, Shamir R, Szajewska H (2008). ESPGHAN/ESPID Evidence-Based Guidelines for the Management of Acute Gastroenteritis in Children in Europe Expert Working Group. European Society for Paediatric Gastroenterology, Hepatology, and Nutrition/European Society for Paediatric Infectious Diseases evidence-based guidelines for the management of acute gastroenteritis in children in Europe: executive summary. J Pediatr Gastroenterol Nutr.

[6] Shornikova AV, Casas IA, Mykkänen H, Salo E, Vesikari T (1997). Bacteriotherapy with Lactobacillus reuteri in rotavirus gastroenteritis. Pediatr Infect Dis J.

[7] Rosander A, Connolly E, Roos S (2008). Removal of antibiotic resistance gene-carrying plasmids from Lactobacillus reuteri ATCC 55730 and characterization of the resulting daughter strain, L. reuteri DSM 17938. Appl Environ Microbiol.

[8] Jones SE, Versalovic J (2009). Probiotic Lactobacillus reuteri biofilms produce antimicrobial and anti-inflammatory factors. BMC Microbiol.

[9] Mu Q, Tavella VJ, Luo XM (2018). Role of Lactobacillus reuteri in Human Health and Diseases. Front Microbiol.

[10] Szymański H, Szajewska H (2019). Lack of efficacy of Lactobacillus reuteri DSM 17938 for the treatment of acute gastroenteritis: a randomized controlled trial. Pediatr Infect Dis J.

[11] Szajewska H, Urbańska M, Chmielewska A, Weizman Z, Shamir R (2014). Meta-analysis: Lactobacillus reuteri strain DSM 17938 (and the original strain ATCC 55730) for treating acute gastroenteritis in children. Benef Microbes.

[12] Akl EA, Altman DG, Aluko P, Askie  LM, Young C (2019). Cochrane Handbook for Systematic Reviews of Interventions.

[13] Maragkoudaki M, Chouliaras G, Moutafi A, Thomas A, Orfanakou A, Papadopoulou A (2018). Efficacy of an oral rehydration solution enriched with Lactobacillus reuteri DSM 17938 and Zinc in the management of Acute Diarrhoea in infants: a randomized, double-blind, placebo-controlled trial. Nutrients.

[14] Shornikova AV, Casas IA, Isolauri E, Mykkänen H, Vesikari T (1997). Lactobacillus reuteri as a therapeutic agent in acute diarrhea in young children. J Pediatr Gastroenterol Nutr.

[15] Dinleyici EC, Dalgic N, Guven S, Metin O, Yasa O, Kurugol Z, Turel O, Tanir G, Yazar AS, Arica V, Sancar M, Karbuz A, Eren M, Ozen M, Kara A, Vandenplas Y (2015). Lactobacillus reuteri DSM 17938 shortens acute infectious diarrhea in a pediatric outpatient setting. J Pediatr (Rio J).

[16] Francavilla R, Lionetti E, Castellaneta S, Ciruzzi F, Indrio F, Masciale A, Fontana C, La Rosa MM, Cavallo L, Francavilla A (2012). Randomised clinical trial: Lactobacillus reuteri DSM 17938 vs. placebo in children with acute diarrhoea–a double-blind study. Aliment Pharmacol Ther.

[17] Pernica JM, Steenhoff AP, Mokomane M, Moorad B, Lechiile K, Smieja M, Mazhani L, Cheng J, Kelly MS, Loeb M, Stordal K, Goldfarb DM (2017). Rapid enteric testing to permit targeted antimicrobial therapy, with and without Lactobacillus reuteri probiotics, for paediatric acute diarrhoeal disease in Botswana: a pilot, randomized, factorial, controlled trial. PLoS One.

[18] Dinleyici EC, Vandenplas Y, PROBAGE Study Group (2014). Lactobacillus reuteri DSM 17938 effectively reduces the duration of acute diarrhoea in hospitalised children. Acta Paediatr.

[19] Wanke M, Szajewska H (2012). Lack of an effect of Lactobacillus reuteri DSM 17938 in preventing nosocomial diarrhea in children: a randomized, double-blind, placebo-controlled trial. J Pediatr.

[20] Urbańska M, Gieruszczak-Białek D, Szymański H, Szajewska H (2016). Effectiveness of Lactobacillus reuteri DSM 17938 for the prevention of Nosocomial diarrhea in children: a randomized, double-blind Placebo-controlled Trial. Pediatr Infect Dis J.

[21] Kołodziej M, Szajewska H (2019). Lactobacillus reuteri DSM 17938 in the prevention of antibiotic-associated diarrhoea in children: a randomized clinical trial. Clin Microbiol Infect.

[22] Dinleyici EC, Kara A, Ozen M, Vandenplas Y (2014). Saccharomyces boulardii CNCM I-745 in different clinical conditions. Expert Opin Biol Ther.

[23] Urbańska M, Gieruszczak-Białek D, Szajewska H (2016). Systematic review with meta-analysis: Lactobacillus reuteri DSM 17938 for diarrhoeal diseases in children. Aliment Pharmacol Ther.

[24] Patro-Gołąb B, Szajewska H (2019). Systematic review with meta-analysis: Lactobacillus reuteri DSM 17938 for treating acute gastroenteritis in children. An Update Nutrients.

[25] Sagheddu V, Uggeri F, Belogi L, Remollino L, Brun P, Bernabè G, Moretti G, Porzionato A, Morelli L, Castagliuolo I, Elli M (2020). The Biotherapeutic Potential of Lactobacillus reuteri characterized using a target-specific selection process. Front Microbiol.

[26] Schnadower D, Tarr PI, Freedman SB (2019). Letter: Lactobacillus rhamnosus GG offers no benefit over placebo in children with acute gastroenteritis. Aliment Pharmacol Ther.

[27] Feizizadeh S, Salehi-Abargouei A, Akbari V (2014). Efficacy and safety of Saccharomyces boulardii for acute diarrhea. Pediatrics.

[28] Padayachee M, Visser J, Viljoen E, Musekiwa A, Blaauw R (2019). Efficacy and safety of Saccharomyces boulardii in the treatment of acute gastroenteritis in the paediatric population: a systematic review. S Afr J Clin Nutr.

[29] Athalye-Jape G, Rao S, Patole S (2016). Lactobacillus reuteri DSM 17938 as a probiotic for preterm neonates: a strain-specific systematic review. JPEN J Parenter Enteral Nutr.

[30] Deng Z, Dai T, Zhang W, Zhu J, Luo XM, Fu D, Liu J, Wang H (2020). Glyceraldehyde-3-Phosphate Dehydrogenase Increases the Adhesion of Lactobacillus reuteri to Host Mucin to Enhance Probiotic Effects. Int J Mol Sci.

[31] Hempel S, Newberry S, Ruelaz A, Wang Z, Miles JN, Suttorp MJ, Johnsen B, Shanman R, Slusser W, Fu N, Smith A, Roth B, Polak J, Motala A, Perry T, Shekelle PG (2011). Safety of probiotics used to reduce risk and prevent or treat disease. Evid Rep Technol Assess (Full Rep).

[32] Schnadower D, Tarr PI, Casper TC, Gorelick MH, Dean JM, O'Connell KJ, Mahajan P, Levine AC, Bhatt SR, Roskind CG, Powell EC, Rogers AJ, Vance C, Sapien RE, Olsen CS, Metheney M, Dickey VP, Hall-Moore C, Freedman SB (2018). Lactobacillus rhamnosus GG versus Placebo for Acute Gastroenteritis in Children. N Engl J Med.

[33] Freedman SB, Williamson-Urquhart S, Farion KJ, Gouin S, Willan AR, Poonai N, Hurley K, Sherman PM, Finkelstein Y, Lee BE, Pang XL, Chui L, Schnadower D, Xie J, Gorelick M, Schuh S, PERC PROGUT Trial Group (2018). Multicenter Trial of a Combination Probiotic for Children with Gastroenteritis. N Engl J Med.

